# A Novel Transdiagnostic Approach to the Prevention of Eating Disorders Using Virtual Reality: Preliminary Evaluation of the H.O.M.E. Intervention

**DOI:** 10.1002/cpp.70040

**Published:** 2025-02-03

**Authors:** Valentina Gardini, Silvana Grandi, Elena Tomba

**Affiliations:** ^1^ Department of Psychology University of Bologna Bologna Italy

**Keywords:** eating disorder prevention, emotion regulation, experiential avoidance, psychological flexibility, transdiagnostic approach, virtual reality

## Abstract

“Virtual reality” (VR) has been used effectively in clinical psychology to improve existing treatments and prevention protocols for many psychopathologies, including eating disorders (EDs). However, no VR software was developed to concurrently tackle dysfunctional eating behaviours and three third wave cognitive–behavioural transdiagnostic factors linked to EDs: psychological inflexibility, emotion dysregulation and experiential avoidance. This preliminary study is aimed at evaluating potential effects of a new VR‐based preventive intervention (H.O.M.E.—How to Observe and Modify Emotions) in improving selected outcomes of transdiagnostic factors and dysfunctional eating behaviours in the general population (GP) with ED risk compared to a waiting list (WL). *N* = 40 GP participants with ED risk were screened (using the SCOFF and Eating Disorder Examination Questionnaire (EDE‐Q)) and randomised into VR (*n* = 20) or WL (n = 20) groups. Before and after intervention and at 3‐ and 6‐month follow‐up, participants completed EDE‐Q, Difficulties in Emotion Regulation Scale—brief version (DERS‐16) for emotion dysregulation, Acceptance and Action Questionnaire II (AAQ‐II) for psychological inflexibility and Multidimensional Psychological Flexibility Inventory (MPFI) experiential avoidance scale. H.O.M.E. improved scores in all EDE‐Q subscales (EDE‐Q‐total: *p* = 0.003; EDE‐Q‐restraint: *p* = 0.028; EDE‐Q‐eating concerns: *p* = 0.035; EDE‐Q‐shape concerns: *p* = 0.003; EDE‐Q‐weight concerns: *p* = 0.023), AAQ‐II (*p* = 0.005), DERS‐16‐total (*p* = 0.006), DERS‐16‐difficulty in engaging in goal‐directed behaviours (*p* = 0.008), and DERS‐16‐limited access to emotion regulation strategies (*p* = 0.001), with results greater than WL and maintained at follow‐ups. Results showed potential for H.O.M.E. to represent a feasible tool to prevent EDs. Given the similarity between VR and technologies used in everyday life, H.O.M.E. may help in engaging young individuals with ED risk towards psychological support before ED onset.

**Summary**

- Virtual reality (VR) demonstrated to be able to enhance treatments for psychological disorders, including eating disorders (EDs), but transdiagnostic VR software are scarce.
- H.O.M.E. (How to Observe and Modify Emotions) is the first VR‐based intervention designed to target dysfunctional eating behaviours and three transdiagnostic factors linked to EDs: psychological inflexibility, emotion dysregulation and experiential avoidance.
- This preliminary study demonstrated that H.O.M.E. managed to improve some of the selected outcomes (i.e., dysfunctional eating behaviours, psychological flexibility and emotion regulation difficulties) in participants with ED risk.
- Preliminary results of the H.O.M.E. VR‐based intervention were greater than the waiting list and maintained at 3‐ and 6‐month follow‐ups.
- These preliminary results show that the H.O.M.E. VR‐based intervention has good feasibility, with no drop‐out rates and promising changes on selected outcomes, but its efficacy needs to be tested in bigger clinical trials with active comparison groups (e.g., CBT‐E).

## 1 Introduction

“Virtual reality” (VR) includes an array of technologies that allow individuals to experience and interact with computer‐generated three‐dimensional environments and objects through a head‐mounted display (Maples‐Keller et al. 2017). The application of VR in clinical psychological contexts as an instrument to offer patients a systematic and controlled exposure therapy without the complications of in vivo exposure (Wiederhold and Wiederhold 2005) has been supported and VR showed to improve existing cognitive–behavioural treatments (CBTs) and prevention protocols especially in anxiety, psychotic, substance‐related and eating disorders (EDs) (Emmelkamp and Meyerbröker 2021; Freeman et al. 2017). Recently, VR programmes capable of being administered across several psychiatric diagnoses have been designed (Colombo et al. 2021). According to the transdiagnostic approach, many psychological disorders and comorbid psychiatric diagnoses are maintained or caused by similar cognitive and/or behavioural processes (Harvey et al. 2004; Mansell et al. 2008), defined as transdiagnostic factors. While research has yet to come to a consensus regarding a fixed number of specific transdiagnostic factors leading to the main psychological disorders, studies showed how, especially within the more recent third wave cognitive behavioural theory of mental disorders (Hayes and Hofmann 2017), several psychiatric diagnoses (e.g., mood and anxiety disorders, substance use disorders and EDs) are all characterised by psychological inflexibility (Hayes et al. 2006), emotion dysregulation (Aldao, Nolen‐Hoeksema, and Schweizer 2010; Fairholme et al. 2010) and experiential avoidance (Chawla and Ostafin 2007; Hayes et al. 1996).

One of the clinical fields in which transdiagnostic approaches found the most evidence is EDs (Atwood and Friedman 2020). According to the main transdiagnostic cognitive–behavioural theoretical model for EDs (Fairburn, Cooper, and Shafran 2003), all EDs are characterised by similar ED features and maintained by shared nonspecific ED factors. Third wave cognitive–behavioural transdiagnostic factors (i.e., psychological inflexibility, emotion dysregulation and experiential avoidance) would also play a role in the onset and maintenance of EDs both in the clinical (Rawal, Park, and Williams 2010; Westwood et al. 2017) and general population (GP) (Della Longa and De Young 2018; Han and Pistole 2014; Masuda et al. 2010). Therefore, their reduction could help improve dysfunctional eating behaviours (Bluett et al. 2016; Holmqvist Larsson et al. 2020; Skinner, Rojas, and Veilleux 2017).

Despite researches supporting both transdiagnostic treatments (Atwood and Friedman 2020) and VR‐based interventions for EDs (Riva, Malighetti, and Serino 2021), a systematic review (Gardini et al. 2022) found that so far no VR software has been designed to concurrently tackle psychological inflexibility, experiential avoidance and emotion regulation (ER). Moreover, no research has focused on the use of VR in the prevention of EDs by targeting these three transdiagnostic factors. Indeed, existing prevention strategies often focus on psychoeducation, cognitive–behavioural techniques and school‐based interventions aimed at addressing body image concerns and reducing eating disorder behaviours (Le et al. 2017; Shaw, Stice, and Becker 2009; Stice, Onipede, and Marti 2021). While traditional prevention approaches have shown efficacy in some populations, they often face limitations, including low engagement rates and motivational barriers (Atkinson and Wade 2013; Harrer et al. 2020; Watson et al. 2016). Using VR in ED prevention may help solve these issues and motivate at‐risk individuals towards change, similar to what has been observed in other populations (Riva 2022). Immersivity and realism offered by VR allow individuals to experience a sense of “emotional presence” comparable to reality in inducing emotional responses (Gorini et al. 2010; Riva, Molinari, and Vincelli 2002) which can be used to provide immersive experiences to at‐risk individuals that could improve the aforementioned transdiagnostic factors linked to EDs.

Therefore, as part of the published H.O.M.E. (How to Observe and Modify Emotions) research protocol (Gardini et al. 2023), this preliminary study had three major aims: (1) to explore the feasibility of a VR‐based intervention conducted using an innovative transdiagnostic VR software (the H.O.M.E. software) (Gardini et al. 2023) in improving dysfunctional eating behaviours and transdiagnostic factors (i.e., ER difficulties, psychological inflexibility and experiential avoidance) between pre‐ and postintervention in the GP at risk for EDs in order to evaluate whether its efficacy can be tested in bigger clinical trials, (2) to observe the potential effects of the H.O.M.E. VR‐based intervention compared to a waiting list condition before moving forward to testing it when used against to or in combination with traditional active psychological interventions (e.g., CBT‐E) and (3) to evaluate whether the changes achieved by the H.O.M.E. VR‐based intervention in the current study were maintained at 3‐ and 6‐month follow‐up.

## 2 Materials and Methods

### 2.1 Ethical Approval

This project was initially approved on 09 October 2020 (Protocol No. 224366) by the Ethical Committee for Psychological Research of University of Bologna and subsequently revised on 14 December 2021 (Protocol No. 0314877).

### 2.2 Recruitment Method

The presented preliminary study investigating the potential effects of the H.O.M.E. VR‐based intervention protocol (Gardini et al. 2023) included individuals from the GP at risk for EDs. Participants were recruited via social media (e.g., Facebook, Instagram). Individuals interested in taking part in the research were informed about aims and characteristics of the study, and informed consent was requested. Inclusion criteria were (a) 18–60 years of age, (b) screening at risk for EDs according to screening instruments reported below, (c) no prior diagnosis of EDs and (d) no health condition that better explains ED symptomatology. Exclusion criteria included (a) lack of capacity to consent for research, (b) current or former ED or other psychiatric diagnosis according to DSM‐5‐TR (American Psychiatric Association 2022) and (c) using medical devices (e.g., pacemaker, hearing aids) or having medical diagnoses (e.g., vertigo, vision impairments) interfering with VR use. Eligible participants were subsequently invited to take part in the study. Those agreeing underwent a brief clinical interview conducted by a clinical psychologist (V.G.) based on DSM‐5‐TR (American Psychiatric Association 2022) criteria to exclude presence of EDs or other psychiatric disorders. The clinical interview was performed before participants took part in the experimental phase of the study (see the Research Design section) and information was provided about their screening results, and if needed, they were informed about the psychological support options available within and outside the research and the contact details of the closest National Health Service ED Centre were provided.

### 2.3 Research Design

The present preliminary study stems from the H.O.M.E. published research protocol (Gardini et al. 2023), which was subsequently divided in three studies conducted on GP participants with ED risk with different aims: (1) to preliminarily explore the potential effects and feasibility of the H.O.M.E. six‐session VR‐based intervention protocol in the improvement of the selected outcomes of dysfunctional eating behaviours and transdiagnostic factors in the GP at risk for EDs, also when compared to a waiting list; (2) to evaluate the quality of the H.O.M.E. software using quantitative measures (e.g., self‐report questionnaires measuring user experience, sense of presence, motion sickness, possible elicited distress) and (3) to evaluate the quality of the H.O.M.E. VR‐based intervention from the participants' point of view using qualitative methods (thematic analysis on interviews conducted on participants completing the protocols).

The presented preliminary study specifically focused on the first aim of the aforementioned research protocol.

To achieve this aim, suitable participants screened as at risk for EDs during a *screening phase* (see the Measures section) were subsequently contacted via e‐mail or telephone within 7 days and invited to take part in the *experimental phase* of the study.

In the *experimental phase*, participants were randomised in the intervention (VR) or control (waiting list) group following a “block randomisation” method (Kim and Shin 2014). Participants in the intervention (VR) group underwent six 30‐min sessions of the VR‐based intervention administered using the software H.O.M.E. at the presence of a clinical psychologist (V.G.) (using the protocol described in Gardini et al. 2023). Controls (waiting list) did not receive any intervention during the study, but they received minimal attention from the clinical psychologists (V.G., E.T.) through phone check‐up sessions monitoring symptoms and general well‐being. They were also offered to receive the VR‐based intervention after the end of the study.

To test whether changes were maintained over time, both groups were recontacted (V.G., E.T.) for *follow‐ups* after 3 and 6 months after conclusion of the experimental phase.

Immediately before the first session of the experimental phase (T0), at conclusion of the experimental phase (immediately after the last VR session or WL check‐up (T1)) and at 3‐ and 6‐month follow‐ups (T2, T3), participants of both groups were asked to complete online (using the platform Qualtrics) a series of psychometric self‐report questionnaires (see the Measures section).

### 2.4 VR Software: H.O.M.E.

**FIGURE 1:**
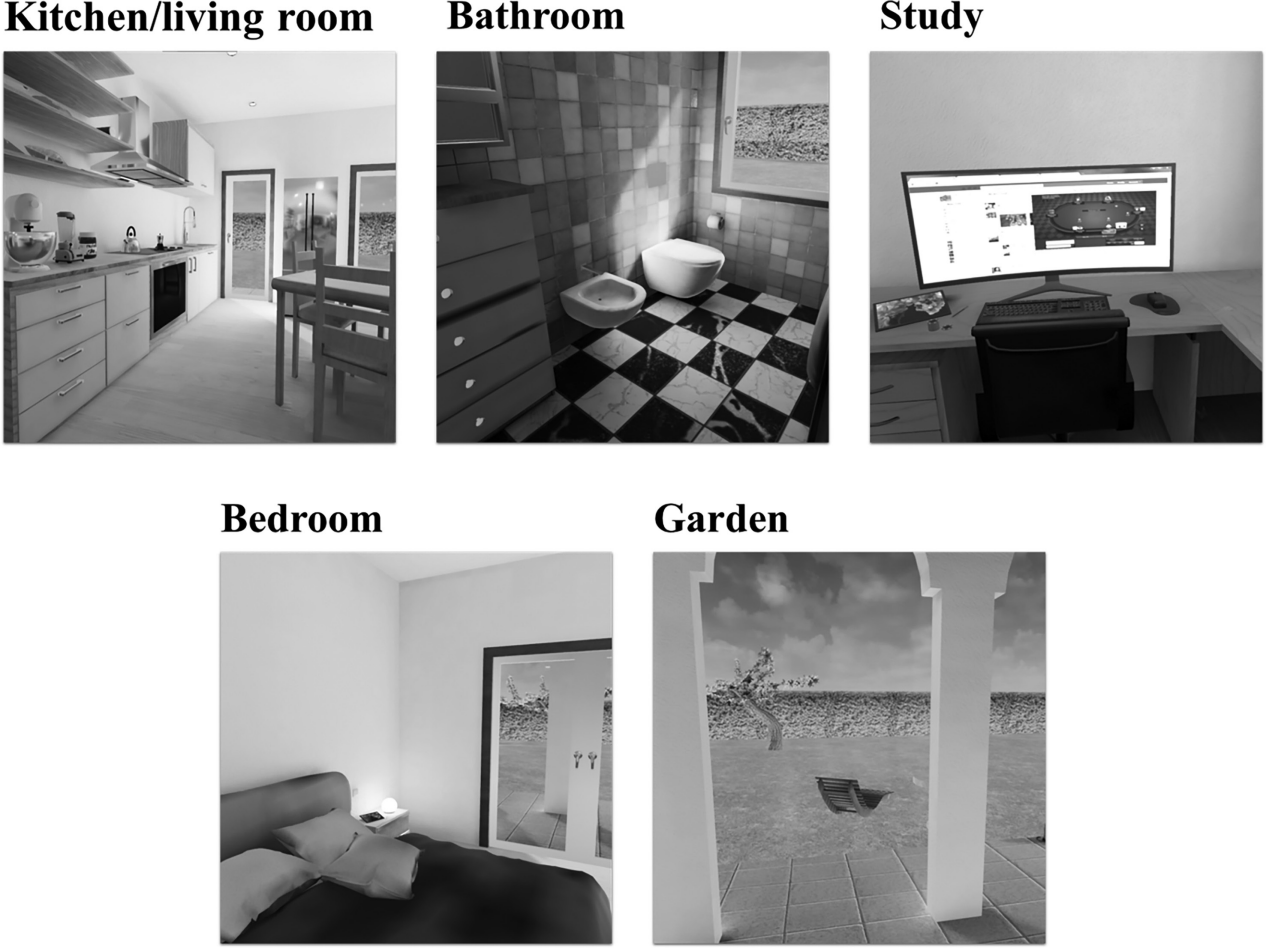
H.O.M.E. software virtual environment (Gardini et al. 2023).

**FIGURE 2:**
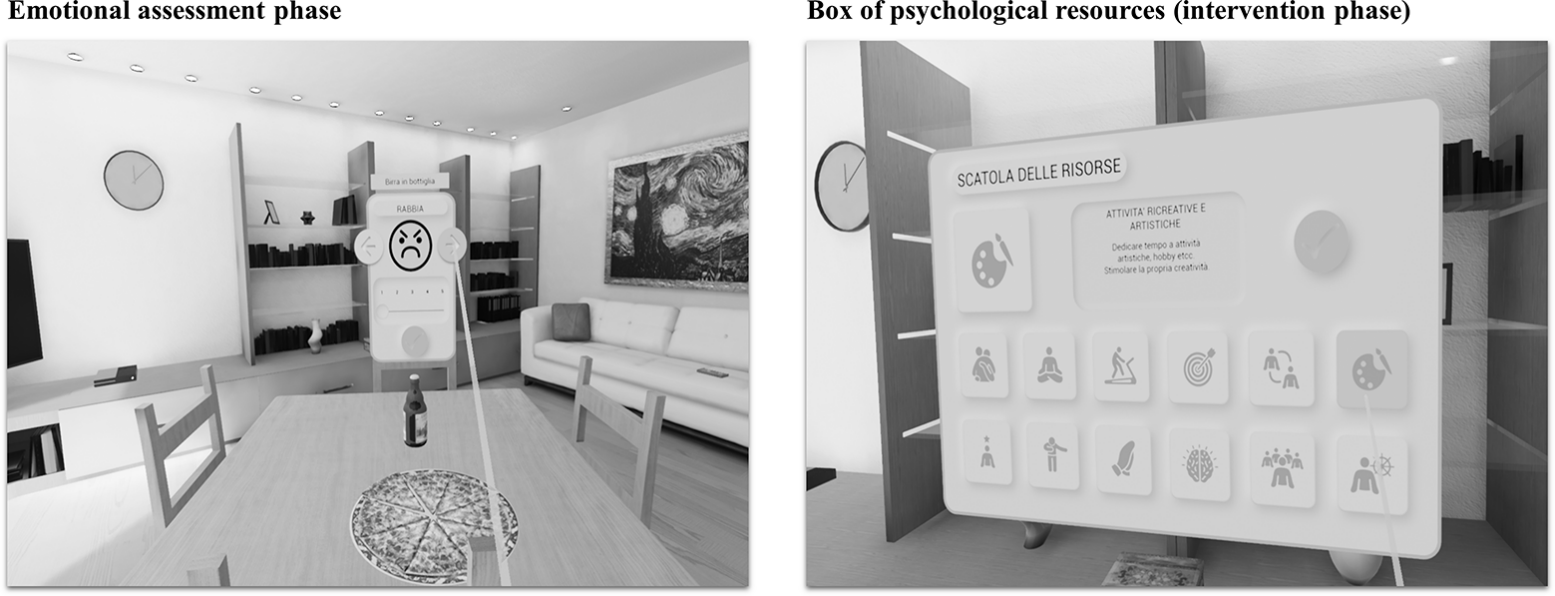
Features available in the H.O.M.E. software (Gardini et al. 2023).

H.O.M.E. is a transdiagnostic VR software developed by a group of clinical psychology researchers from the Department of Psychology of University of Bologna (Gardini et al. 2023). H.O.M.E. can be used by connecting a computer to an Oculus Quest headset. The software allows users to move around a virtual environment consisting of a house with four rooms (i.e., kitchen/living room, bathroom, bedroom and a study) and a garden (Figure 1) and to interact with objects relevant for specific mental health issues (e.g., comfort foods for people with eating‐related issues; alcohol, pills and cigarettes for addictions; a computer with a gambling interface for gambling addictions; videogames and smartphones for technology‐related addictions). The software includes two phases (Figure 2): an assessment phase where users can report the emotion they associate to each object and its intensity and an intervention phase based on the cognitive–behavioural model in which users can access a ‘box of psychological resources’ including pictures and descriptions of emotional, social and behavioural resources that can be used to face the emotions previously attributed to the objects. The software is meant to be transdiagnostic since it is designed to tackle the following transdiagnostic factors: ER strategies, psychological flexibility and emotional avoidance; and it is meant to be used in the prevention and treatment of several psychiatric disorders (e.g., EDs, addictions, substance use disorders) under the instructions of a clinical psychologist. In the present study, objects that could represent a trigger for people at risk for EDs (e.g., food in the kitchen area) were predominantly used.

**FIGURE 3:**
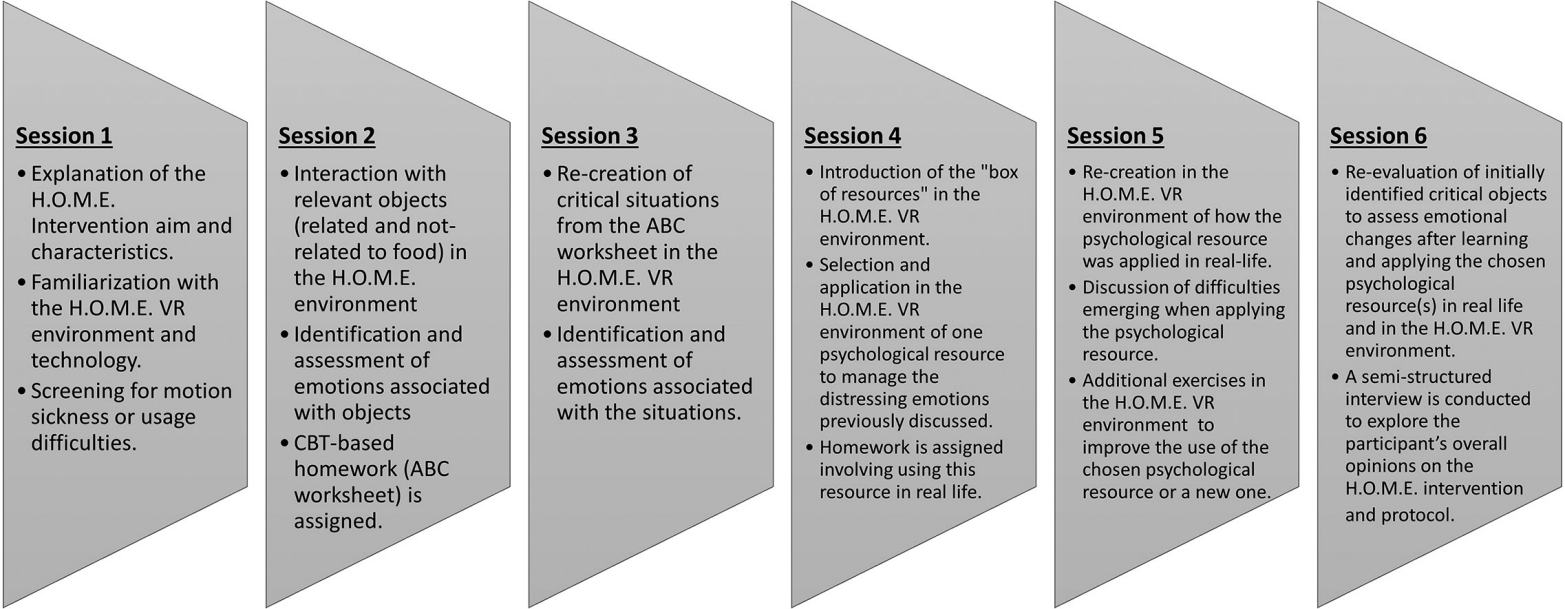
Brief summary of the content and aims of the H.O.M.E. VR‐based intervention sessions (Gardini et al. 2023).

The characteristics and aims of the H.O.M.E. software as well as the content of the H.O.M.E. six‐session VR‐based intervention used for the present study were published in a separate article (Gardini et al. 2023) and are summarised in Figure 3.

### 2.5 Measures

To detect ED risk, recruited GP participants completed the following screening questionnaires:

- A nonstandardised *self‐report questionnaire* to collect sociodemographic (age, marital status, educational level, occupational status) and clinical data (body mass index (BMI), former ED or another DSM‐5 diagnosis, clinical conditions and/or medical devices interfering with VR) and to investigate participants' daily use of technological devices (e.g., computer, videogames)
- The *SCOFF questionnaire* (Morgan, Reid, and Lacey 1999; Pannocchia et al. 2011), a psychometric five‐item self‐report screening questionnaire detecting ED risk. A cut‐off of > 2 indicates a possible ED case (Cronbach's *α* = 0.64 in Italian version)
- The *Eating Disorder Examination Questionnaire* (EDE‐Q 6.0) (Calugi et al. 2017; Fairburn and Beglin 1994). The EDE‐Q produces a global score and four subscales for the past 28 days: eating concern, shape concern, weight concern and restraint (Cronbach's *α* = 0.70–0.83 for subscale; ≥ 0.90 for the global score) (Peterson et al. 2007). Scores above 1.56 are associated with ED risk in the GP (Ekeroth and Birgegård 2014)

A brief clinical interview conducted by a clinical psychologist (V.G.) based on DSM‐5‐TR (American Psychiatric Association 2022) criteria was also performed before the experimental phase of the study on people screening as at risk at the SCOFF and EDE‐Q to exclude presence of EDs or other DSM‐5‐TR psychiatric disorders.

At T0, T1, T2 and T3, recruited participants undergoing the VR‐based interventions or waiting list filled in the following psychometric questionnaires to evaluate changes in dysfunctional eating behaviours and transdiagnostic factors:

- The EDE‐Q 6.0 (Calugi et al. 2017; Fairburn and Beglin 1994)
- The *Difficulties in Emotion Regulation Scale*—*brief version* (DERS‐16) (Bjureberg et al. 2016), a 16‐item self‐report questionnaire evaluating five ER difficulties: nonacceptance of emotional responses (DERS‐16‐NA), difficulty in engaging in goal‐directed behaviours (DERS‐16‐GO), impulse control difficulties (DERS‐16‐IMP), limited access to ER strategies (DERS‐16‐STR) and lack of emotional clarity (DERS‐16‐CL) (Cronbach's *α* = 0.87–0.96) (Hallion et al. 2018)
- The *Acceptance and Action Questionnaire II* (AAQ‐II) (Bond et al. 2011; Pennato et al. 2013), a seven‐item self‐report questionnaire assessing psychological flexibility (Cronbach's *α* = 0.83) (Pennato et al. 2013)
- The *experiential avoidance scale* (MPFI‐EA) from the *Multidimensional Psychological Flexibility Inventory* (MPFI) (Landi et al. 2021; Rolffs, Rogge, and Wilson 2018), a five‐item self‐report scale evaluating experiential avoidance (Cronbach's *α* = 0.91 for Italian version)

### 2.6 Data Analyses

Data analyses were performed using SPSS Statistic vrs.25. Descriptive statistics were run to analyse sociodemographic (age, marital status, educational level, occupational status) and clinical characteristics (weight history, former ED diagnosis, etc.), as well as mean SCOFF, EDE‐Q, DERS‐16, AAQ‐II and MPFI‐EA. The *t*‐tests for continuous variables and chi‐square tests for categorical variables were performed to test differences between those who completed the screening questionnaire and did not accept to take part in the research and those who accepted.

The *t*‐tests and chi‐square tests were also used to test preintervention/baseline differences between experimental (VR) and control (waiting list) groups.

To test the feasibility of the H.O.M.E. VR‐based intervention, percentage of drop‐out cases was calculated for the experimental (VR) group.

To test the potential effects of the H.O.M.E. VR‐based intervention and to also compare its effects (in terms of changes produced in EDE‐Q, DERS‐16, AAQ‐II and MPFI‐EA between pre‐ and postintervention and between postintervention and follow‐ups) to those of the control (waiting list) condition, repeated‐measures ANOVAs were performed using time (T0, T1, T2, T3) as within‐subject factor and group (VR or waiting list) as between‐subject factor with post hoc contrast analyses. Given the preliminary and exploratory nature of the study, multiple comparison corrections (e.g., Bonferroni correction) were not performed (Rothman 1990) and effect sizes (Cohen's *d*) were reported to better understand the magnitude of the potential changes achieved.

## 3 Results

### 3.1 Description of the Sample

*N* = 189 people from the GP completed the online screening questionnaires on the platform Qualtrics. Of these, the number of people at risk for EDs was determined based on the *SCOFF Questionnaire* (Morgan, Reid, and Lacey 1999; Pannocchia et al. 2011) and EDE‐Q 6.0 (Calugi et al. 2017; Fairburn and Beglin 2008) cut‐offs of < 2 and < 1.56 (Ekeroth and Birgegård 2014), respectively. A total of *n* = 104 (55.03%) people screened with ED risk were all contacted to take part in the research. According to the brief clinical interview based on DSM‐5‐TR criteria, *n* = 8 (7.69%) people met criteria for an ED diagnosis and *n* = 12 (11.54%) for other DSM‐5‐TR diagnoses and were excluded from the present study. *N* = 47 people (45.19%), instead, refused to take part in the research for personal or logistical reasons. The final sample included *n* = 40 (38.46%) eligible people at risk for EDs that accepted to take part in the study and were randomised to the experimental (undergoing the H.O.M.E. VR‐based preventive intervention protocol) (*n* = 20) or control (waiting list) (*n* = 20) group using a block randomisation method.

Descriptive analyses revealed that *n* = 64 participants that did not take part in the study were females, with a mean age of 23.6 ± 8.08. Mean SCOFF score was 3.33 ± 0.99, while mean EDE‐Q score was 3.20 ± 1.54. Independent sample *t*‐test revealed that there was no significant difference between those deciding to take part in the research and those who did not participate (see Table S1).

When considering the sample that took part in the research, both the experimental (VR) and control (waiting list) groups were made up of only female participants, with similar mean ages (VR: 24.35 ± 6.02; waiting list: 24.75 ± 9.08). The majority of the sample in both groups were single (VR: *n* = 18, 90%; waiting list: *n* = 18, 90%) and university students (VR: *n* = 15, 75%; waiting list: *n* = 16, 80%). All participants in both groups were familiar with the use of technology (e.g., smartphones, computers, videogames) in their daily lives.

When considering dysfunctional eating behaviours, participants of both groups reported the highest scores in the EDE‐Q‐shape concerns (VR: 4.52 ± 0.93; waiting list: 4.27 ± 1.26) and EDE‐Q‐weight concerns (VR: 3.92 ± 1.11; waiting list: 3.30 ± 1.40) subscales.

Table 1 summarises sociodemographic and clinical characteristics of both groups, as well as the statistical indexes relative to the independent sample *t*‐test and chi‐square tests run to evaluate the presence of possible sociodemographic or clinical differences between groups at baseline (T0).

**TABLE 1:** Sociodemographic and clinical characteristics of the VR and waiting list group samples and differences between groups.

	H.O.M.E. VR group (*n* = 20) *M* ± SD *or* % (*n*=)	Waiting list group (*n* = 20) *M* ± SD *or* % (*n*=)	*t* (df)	*χ* ^2^_(df)_	*p*
Gender	100% (*n* = 20) females	100% (*n* = 20) females	—	—	—
Age	24.35 ± 6.02 years	24.75 ± 9.08 years	−0.16_(38.0)_	—	0.870
Marital status	90.0% (*n* = 18) single 5% (*n* = 1) married 5% (*n* = 1) divorced	90.0% (*n* = 18) single 5% (*n* = 1) married 5% (*n* = 1) divorced	—	—	—
Educational level	35% (*n* = 7) high school diploma 65% (*n* = 13) university degree	50% (*n* = 10) high school diploma 50% (*n* = 10) university degree	—	2.39_(2)_	0.303
Occupation	75% (*n* = 15) university students 20% (*n* = 4) employed 5% (*n* = 1) unemployed	80% (*n* = 16) university students 20% (*n* = 4) employed	—	3.03_(4)_	0.552
BMI	25.72 ± 3.19	22.93 ± 3.28	3.33_(38.0)_	—	0.087
SCOFF	3.65 ± 1.09	3.55 ± 0.95	0.31_(38.0)_	—	0.758
EDE‐Q‐total	3.48 ± 1.07	3.18 ± 1.18	0.85_(38.0)_	—	0.400
EDE‐Q‐restraint	2.79 ± 1.68	2.75 ± 1.38	0.08_(38.0)_	—	0.935
EDE‐Q‐eating concerns	2.69 ± 1.44	2.36 ± 1.33	0.75_(38.0)_	—	0.456
EDE‐Q‐shape concerns	4.52 ± 0.93	4.27 ± 1.26	0.72_(38.0)_	—	0.479
EDE‐Q‐weight concerns	3.92 ± 1.11	3.3 ± 1.40	1.48_(38.0)_	—	0.147
DERS‐16‐total	47.45 ± 15.77	46.95 ± 18.42	0.09_(38.0)_	—	0.927
DERS‐16‐*clarity*	5.75 ± 2.34	5.15 ± 2.76	0.74_(38.0)_	—	0.462
DERS‐16*‐goals*	10.35 ± 3.30	9.80 ± 3.82	0.49_(38.0)_	—	0.629
DERS‐16‐*impulse*	7.55 ± 3.96	7.95 ± 3.76	−0.33_(38.0)_	—	0.745
DERS‐16‐*strategies*	14.70 ± 5.56	15.10 ± 6.50	−0.21_(38.0)_	—	0.836
DERS‐16‐*nonacceptance*	9.10 ± 3.14	8.95 ± 3.94	0.13_(38.0)_	—	0.895
AAQ‐II	28.15 ± 9.56	25.85 ± 10.97	0.71_(38.0)_	—	0.484
MPFI‐EA	3.28 ± 0.99	3.34 ± 1.10	−0.18_(38.0)_	—	0.852

Abbreviations: AAQ‐II, Acceptance and Action Questionnaire II; BMI, body mass index; DERS‐16, Difficulties in Emotion Regulation Scale, short version; EDE‐Q, Eating Disorder Examination Questionnaire; M, mean; MPFI‐EA, Multidimensional Psychological Flexibility Inventory‐experiential avoidance scale; SD, standard deviation.

As it can be observed from Table 1, no significant difference was found between groups at baseline in any of the variables, including dysfunctional eating behaviours (SCOFF, EDE‐Q) and transdiagnostic factors (DERS‐16, AAQ‐II, MPFI‐EA).

### 3.2 Feasibility and Effects of the H.O.M.E. Intervention and Comparison With Waiting List

The H.O.M.E. VR‐based prevention intervention showed good feasibility, with no participant dropping out after undergoing at least one session of the intervention.

These preliminary results showed that the H.O.M.E. VR‐based intervention was also capable of producing changes between pre‐ and postintervention (T0–T1 changes) in participants undergoing it. In particular, mixed‐model repeated‐measures ANOVAs with post hoc contrast revealed significant improvements and/or medium effect size differences between T0 and T1 in EDE‐Q‐total (T0 = 3.48 ± 1.07, T1 = 2.89 ± 1.02, *p* = 0.003, *d* = 0.56) and all EDEQ subscales (EDE‐Q‐restraint: T0 = 2.79 ± 1.68, T1 = 2.22 ± 1.19, *p* = 0.028, *d* = 0.39; EDE‐Q‐eating concerns: T0 = 2.69 ± 1.44, T1 = 2.20 ± 1.22, *p* = 0.035, *d* = 0.37; EDE‐Q‐shape concerns: T0 = 4.52 ± 0.93, T1 = 3.74 ± 1.26, *p* = 0.003, *d* = 0.70; EDE‐Q‐weight concerns: T0 = 3.92 ± 1.11, T1 = 3.38 ± 1.18, *p* = 0.023, *d* = 0.47), AAQ‐II (T0 = 28.15 ± 9.56, T1 = 24.35 ± 10.43, *p* = 0.005, *d* = 0.38), DERS‐16‐total (T0 = 47.45 ± 15.77, T1 = 41.20 ± 16.20, *p* = 0.006, *d* = 0.39), DERS‐16‐difficulty in engaging in goal‐directed behaviours (T0 = 10.35 ± 3.30, T1 = 9.10 ± 3.68, *p* = 0.008, *d* = 0.36) and DERS‐16‐limited access to ER strategies (T0 = 14.70 ± 5.56, T1 = 12.30 ± 5.20, *p* = 0.001, *d* = 0.45) which were observed. Changes achieved at postintervention (T1) were maintained at both 3‐ (T2) and 6‐month (T3) follow‐ups, with a lack of significant and/or medium effect size differences between T1–T2 and T1–T3 in all the improved aforementioned variables, with the only exception of DERS‐16‐difficulty in engaging in goal‐directed behaviours (T1 = 9.10 ± 3.68, T3 = 7.85 ± 3.31, *p* = 0.008, *d* = 0.36) and DERS‐16‐nonacceptance of emotional responses (T1 = 8.40 ± 3.49, T3 = 7 ± 3.28, *p* = 0.006, *d* = 0.41) which improved between postintervention (T1) and 6‐month follow‐up (T3).

Table S2 shows mean levels of dysfunctional eating behaviours (EDE‐Q) and transdiagnostic factors (DERS‐16, AAQ‐II and MPFI‐EA) at each assessment point, separately for each group.

Results from repeated‐measures mixed‐model ANOVAs and statistical data are summarised in Tables 2 and 3.

**TABLE 2:** Results of the repeated‐measures mixed‐model ANOVAs investigating changes in the H.O.M.E. intervention and comparisons with waiting list (EDEQ, AAQ‐II, MPFI‐EA).

		EDEQ‐total	EDEQ‐restraint	EDEQ‐eating concerns	EDEQ‐shape concerns	EDEQ‐weight concerns	AAQ‐II‐total	MPFI‐EA‐total
	Group (*F*_(df)_; *p*; *η* ^2^_p_)	0.76_(38.1)_; 0.389; 0.02	1.77_(38.1)_; 0.191; 0.04	0.30_(38.1)_; 0.589; 0.01	1.05_(38.1)_; 0.313; 0.03	0.010_(38.1)_; 0.919; 0.00	0.49_(38.1)_; 0.487; 0.01	2.12_(38.1)_; 0.153; 0.05
	Time (*F*_(df)_; *p*; *η* ^2^_p_)	2.32_(38.1)_; 0.079; 0.06	1.39_(38.1)_; 0.249; 0.045	2.48_(38.1)_; 0.065; 0.06	**2.98**_**(38.1)**_ **; 0.035; 0.07**	0.91_(38.1)_; 0.440; 0.02	1.31_(38.1)_; 0.275; 0.03	1.15_(38.1)_; 0.333; 0.03
	Time*group (*F*_(df)_; *p*; *η* ^2^_p_)	**5.37**_**(38.1)**_ **; 0.002; 0.12**	**2.00**_**(38.1)**_ **; 0.017; 0.09**	**3.84**_**(38.1)**_ **; 0.012; 0.09**	**3.58**_**(38.1)**_ **; 0.016; 0.09**	**3.54**_**(38.1)**_ **; 0.017; 0.09**	**5.90**_**(38.1)**_ **; < 0.001; 0.13**	0.88_(38.1)_; 0.452; 0.02
*Post hoc*								
VR MD (*p*; d)	T0 × T1	**0.59 (0.003; 0.56)**	**0.57 (0.028; 0.39)**	**0.49 (0.035; 0.37)**	**0.78 (0.003; 0.70)**	**0.54 (0.023; 0.47)**	**3.80 (0.005; 0.38)**	−0.12 (0.679; −0.11)
	T0 × T2	**0.67 (0.002; 0.54)**	0.42 (0.098; 0.27)	**0.71 (0.002; 0.47)**	**0.87 (0.006; 0.61)**	**0.67 (0.013; 0.47)**	**4.40 (0.005; 0.41)**	0.20 (0.545; 0.18)
	T0 × T3	**0.62 (0.005; 0.54)**	0.49 (0.092; 0.33)	**0.69 (0.005; 0.49)**	**0.81 (0.004; 0.64)**	0.51 (0.062; 0.41)	**3.85 (0.017; 0.37)**	0.28 (0.367; 0.26)
	T1 × T2	0.07 (0.657; 0.07)	−0.15 (0.507; −0.12)	0.22 (0.176; 0.16)	0.09 (0.679; 0.06)	0.13 (0.545; 0.09)	0.60 (0.551; 0.05)	0.32 (0.208; 0.27)
	T1 × T3	0.03 (0.381; 0.03)	−0.08 (0.732; −0.07)	0.20 (0.310; 0.16)	0.03 (0.866; 0.02)	−0.03 (0.888; −0.02)	0.05 (0.968; 0.01)	0.40 (0.094; 0.35)
	T2 × T3	−0.04 (0.683; −0.04)	0.07 (0.702; 0.05)	−0.02 (0.848; −0.01)	−0.06 (0.544; −0.04)	−0.16 (0.293; −0.10)	−0.55 (0.606; −0.05)	0.08 (0.428; 0.07)
Waiting listMD (*p*; *d*)	T0 × T1	−0.13 (0.492; −0.11)	−0.10 (0.690; −0.08)	−0.10 (0.658; −0.08)	−0.06 (0.799; −0.05)	−0.26 (0.260; −0.20)	−1.40 (0.274; −0.13)	−0.45 (0.126; −0.41)
	T0 × T2	−0.04 (0.848; −0.03)	−0.02 (0.936; −0.01)	0.02 (0.927; 0.02)	0.02 (0.950; 0.02)	−0.17 (0.512; −0.14)	−1.30 (0.385; −0.12)	−0.20 (0.545; −0.19)
	T0 × T3	−0.31 (0.154; −0.28)	−58 (0.047; −0.44)	−0.19 (0.415; −0.15)	−0.09 (0.727; −0.07)	−0.37 (0.172; −0.29)	**−3.25 (0.042; −0.32)**	−0.31 (0.318; −0.29)
	T1 × T2	0.09 (0.575; 0.09)	0.08 (0.723; 0.06)	0.22 (0.176; 0.10)	0.08 (0.720; 0.06)	0.09 (0.675; 0.06)	0.10 (0.921; 0.01)	0.25 (0.323; 0.24)
	T1 × T3	−0.18 (0.296; −0.17)	**−0.48 (0.046; −0.38)**	−0.35 (0.389; −0.07)	−0.03 (0.866; −0.02)	−0.11 (0.607; −0.08)	−1.85 (0.143; −0.19)	0.14 (0.552; 0.13)
	T2 × T3	**−0.27 (0.014; −0.26)**	**−0.56 (0.004; −0.42)**	−0.57 (0.180; −0.18)	−0.11 (0.277; −0.08)	−0.20 (0.190; −0.14)	−1.95 (0.073; −0.20)	−0.11 (0.278; −0.11)

*Note:* Bold: *p* < 0.05. Abbreviations: AAQ‐II, Acceptance and Action Questionnaire II; EDE‐Q, Eating Disorder Examination Questionnaire; MD, mean difference; MPFI‐EA, Multidimensional Psychological Flexibility Inventory‐experiential avoidance scale.

**TABLE 3:** Results of the repeated‐measures mixed‐model ANOVAs investigating changes in the H.O.M.E. intervention and comparisons with waiting list (DERS‐16).

		DERS‐16‐total	DERS‐16‐*lack of emotional clarity*	DERS‐16‐*difficulty in engaging in goal directed behaviours*	DERS‐16‐*impulse control difficulties*	DERS‐16‐*limited access to emotion regulation strategies*	DERS‐16‐*nonacceptance of emotional responses*
	Group (*F*_(df)_; *p*; *η* ^2^_p_)	2.41_(38.1)_; 0.129; 0.06	0.23_(38.1)_; 0.632; 0.01	2.42_(38.1)_; 0.128; 0.06	1.62_(38.1)_; 0.211; 0.04	2.70_(38.1)_; 0.108; 0.07	1.90_(38.1)_; 0.176; 0.05
	Time (*F*_(df)_; *p*; *η* ^2^_p_)	**2.92**_**(38.1)**_ **; 0.037; 0.07**	0.80_(38.1)_; 0.495; 0.02	1.88_(38.1)_; 0.137; 0.05	2.40_(38.1)_; 0.072; 0.06	**2.81**_**(38.1)**_ **; 0.043; 0.07**	**2.81**_**(38.1)**_ **; 0.043; 0.07**
	Time*group (*F*_(df)_; *p*; *η* ^2^_p_)	**9.63**_**(38.1)**_ **; < 0.001; 0.20**	2.40_(38.1)_; 0.071; 0.06	13.52_**(38.1)**_ **; < 0.001; 0.26**	2.53_(38.1)_; 0.061; 0.06	**7.64; < 0.001; 0.17**	7.50_**(38.1)**_ **; < 0.001; 0.17**
*Post hoc*							
VR MD (*p*; d)	T0 × T1	**6.25 (0.006; 0.39)**	0.85 (0.080; 0.38)	**1.25 (0.008; 0.36)**	1.05 (0.072; 0.26)	**2.40 (0.001; 0.45)**	0.70 (0.204; 0.21)
	T0 × T2	**8.70 (< 0.001; 0.53)**	0.75 (0.844; 0.30)	**2.05 (< 0.001; 0.61)**	**1.35 (0.023; 0.36)**	**2.60 (0.005; 0.44)**	**1.95 (< 0.001; 0.60)**
	T0 × T3	**10.00 (< 0.001; 0.62)**	0.55 (0.249; 0.23)	**2.50 (< 0.001; 0.76)**	**1.60 (0.004; 0.43)**	**3.25 (< 0.001; 0.56)**	**2.10 (< 0.001; 0.65)**
	T1 × T2	2.45 (0.220; 0.15)	−0.10 (0.805; −0.04)	0.80 (0.056; 0.23)	0.30 (0.537; 0.08)	0.20 (0.759; 0.04)	**1.25 (0.014; 0.36)**
	T1 × T3	3.75 (0.061; 0.23)	−0.30 (0.426; −0.13)	**1.25 (0.008; 0.36)**	0.55 (0.319; 0.14)	0.85 (0.229; 0.15)	**1.40 (0.006; 0.41)**
	T2 × T3	1.30 (0.283; 0.08)	−0.20 (0.400; −0.08)	0.45 (0.148; 0.13)	0.25 (0.328; 0.07)	0.65 (0.137; 0.11)	0.15 (0.591; 0.05)
Waiting listMD (*p*; *d*)	T0 × T1	−0.80 (0.712; −0.05)	−0.45 (0.347; −0.18)	−0.50 (0.269; −0.14)	0.20 (0.726; 0.06)	0.10 (0.885; 0.02)	−0.15 (0.783; −0.04)
	T0 × T2	−0.30 (0.901; −0.02)	−0.25 (0.604; −0.10)	−0.50 (0.181; −0.14)	0.50 (0.385; 0.14)	0.00 (1.00; < 0.001)	−0.05 (0.923; −0.01)
	T0 × T3	**−5.35 (0.028; −0.34)**	−0.85 (0.078; −0.34)	**−0.1.60 (0.008; −0.49)**	−0.35 (0.511; −0.10)	−1.60 (0.075; −0.28)	−0.95 (0.102; −0.28)
	T1 × T2	0.50 (0.801; 0.03)	0.20 (0.622; 0.09)	0.00 (1.00; < 0.001)	0.30 (0.537; 0.10)	−0.10 (0.878; −0.02)	0.10 (0.837; 0.03)
	T1 × T3	**−4.55 (0.025; −0.33)**	−0.40 (0.583; −0.18)	**−1.10 (0.018; −0.35)**	−0.55 (0.319; −0.18)	**−1.70 (0.019; −0.31)**	−0.80 (0.103; −0.27)
	T2 × T3	**−5.05 (< 0.001; −0.36)**	**−0.60 (0.015; −0.27)**	**−3.10 (0.004; −0.36)**	**−0.85 (0.002; −0.28)**	**−1.60 (< 0.001; −0.30)**	**−0.90 (0.002; −0.31)**

*Note:* Bold: *p* < 0.05. Abbreviations: DERS‐16, Difficulties in Emotion Regulation Scale, short version; MD, mean difference.

Looking at the results from the ANOVAs, all the changes reported by the H.O.M.E. VR‐based intervention also resulted to be greater than those of the waiting list condition (significant time × group interactions, *p* levels and effect sizes are reported in Tables 2 and 3).

The waiting list also did not report any significant improvement in dysfunctional eating behaviours or any of the transdiagnostic factors between T0 and T1, but levels of EDE‐Q‐restraint (T1 = 2.85 ± 1.27, T3 = 3.33 ± 1.23, *p* = 0.046, *d* = −0.38), DERS‐16‐total (T1 = 47.75 ± 15.13, T3 = 52.30 ± 12.76, *p* = 0.025, *d* = −0.33), DERS‐16‐difficulty in engaging in goal‐directed behaviours (T1 = 10.30 ± 3.47, T3 = 11.40 ± 2.70, *p* = 0.018, *d* = −0.35) and DERS‐16‐limited access to ER strategies (T1 = 15 ± 5.91, T3 = 16.70 ± 4.95, *p* = 0.019, *d* = −31) even worsened between T1 and 6‐month (T3) follow‐up as represented by significant T1–T3 differences.

## 4 Discussion

This preliminary study was part of the published H.O.M.E. research protocol (Gardini et al. 2023), and it is aimed at exploring the feasibility and potential effects of the transdiagnostic VR‐based preventive intervention run through the H.O.M.E. software in improving the selected outcomes of transdiagnostic factors (i.e., psychological inflexibility, emotion dysregulation and experiential avoidance) and dysfunctional eating behaviours in the GP with ED risk also when compared to a waiting list. This study was conducted in order to explore and evaluate whether the H.O.M.E. VR‐based intervention protocol has potential to be tested in bigger clinical trials and when used against to or in combination with active traditional psychological interventions (e.g., CBT‐E).

Through a battery of online self‐report screening questionnaires, people from the GP at risk of developing EDs were detected. However, a wide range of respondents that were contacted decided not to take part in the intervention. This is not surprising, as people suffering from EDs are often reluctant or motivated to seek help, due to the egosyntonic nature of these psychopathologies especially in the early stages (Roncero et al. 2013).

Regarding the sample of people who accepted to take part in the study, it consisted of mainly single women and university students, which is unsurprising considering that young women are more frequently affected by EDs (Jacobi et al. 2004). These sociodemographic characteristics are also consistent with samples of prevention interventions, which are often targeted at young adults (Watson et al. 2016). In fact, epidemiological data underline that it is precisely this segment of the population that is most at risk of developing EDs (Mastrobattista and Pacifici 2022) and, therefore, it is necessary to identify effective preventive intervention strategies that can be carried out on this population (Schwartz et al. 2019).

The importance of body and weight in today's society, which encourages young women in particular to pursue an ideal of thinness often unhealthy (Culbert, Racine, and Klump 2015), is reflected in the high levels of body shape concerns and weight concerns measured through the EDE‐Q, which were the highest subscales in all samples. The levels of difficulties in ER and psychological inflexibility reported by participants were also higher than in other studies conducted on the GP without ED risk (McClure et al. 2022; Pennato et al. 2013). Indeed, as underlined by the literature (Morton et al. 2020; Prefit, Cândea, and Szentagotai‐Tătar 2019), dysfunctional eating behaviours are often associated with difficulties in ER and psychological inflexibility. Levels of experiential avoidance, instead, were consistent with GP levels found in the literature (Landi et al. 2021).

Regarding the specific results obtained by this preliminary study, the H.O.M.E. VR‐based preventive intervention showed promising results in improving all dysfunctional eating behaviours in the sample with significant differences and/or medium effect size differences between pre‐ and postintervention in the VR group. Levels of concerns regarding body shape were the variables that showed the greatest improvement (in terms of the greatest mean difference and effect size difference reported between pre‐ and postintervention scores). This scale was also the ED‐related symptom with the highest levels in the sample at baseline. The improvement reported after the H.O.M.E. intervention is in line with results from other studies, which found that body shape concerns represent one of the ED‐related symptoms which can more easily be improved by VR‐based interventions, especially when VR is offered in addition to CBT‐based protocols (Freeman et al. 2017). Similar to the presented preliminary results, reductions in the levels of concerns about eating and weight have also been found in the literature about VR (Irvine et al. 2020). For example, in a study by Irvine et al. (2020), a similar sample of young women with high levels of body image concerns (but without any ED diagnosis) reported improvements in concerns about weight, shape and eating after undergoing a VR‐based training intervention programme through the use of an avatar. Even though the H.O.M.E. software does not offer an avatar feature, our VR‐based intervention was still capable of producing beneficial results, which highlights that H.O.M.E. could have the potential to represent a helpful tool for the prevention of EDs and the reduction of dysfunctional eating behaviours in the GP at risk. Future research is, however, needed in order to confirm these results using more robust methodological approaches and bigger sample sizes.

Concerning the preliminary results about the transdiagnostic factors, as expected, difficulties in ER and psychological inflexibility also showed a significant improvement although with lower effect size differences at postintervention. Instead, no significant change and/or medium effect size differences were found for experiential avoidance.

Starting from the difficulties in ER, this transdiagnostic factor significantly improved both on a general level and in terms of improvements in participants' specific abilities to engage in goal‐directed behaviours and to access their ER strategies. Although other difficulties in ER subscales did not show any improvements, possibly due to the small number of sessions of the intervention or the specific characteristics and targets of the intervention protocol, which mainly focused on helping people select strategies to face distressing emotions, this result brings up some potential beneficial effects of the H.O.M.E. VR‐based preventive intervention. The ability of VR interventions to improve ER in a wide variety of clinical and nonclinical populations is also supported by studies using other VR software and protocols (Hadley et al. 2019). This may depend on the emotional involvement that VR is able to elicit (Colombo et al. 2021). In fact, during the H.O.M.E. VR‐based intervention, participants were exposed to emotional stimuli, such as food, and this allowed an introspective observation of the emotions experienced and their association with dysfunctional eating behaviours. While these exploratory results are encouraging and pave the way for future research, the lack of large effect size differences underlines the need for future more robust studies to truly test the H.O.M.E. VR‐based intervention protocol efficacy and to also improve the current intervention protocol.

Unfortunately, to the best of our knowledge, this preliminary study was also the first in the literature to test the application of a VR intervention for the improvement of psychological inflexibility and experiential avoidance. Even though VR was applied in EDs to improve *body image flexibility*, producing significant changes (Marco, Perpiñá, and Botella 2013), these results showed that VR also holds potential to produce significant improvements in psychological inflexibility but not in experiential avoidance, which could be due both to limitations present in the tool used to assess changes (the MPFI‐EA, which is composed of only five items) and in characteristics of the H.O.M.E. intervention protocol (e.g., short duration). Indeed, these preliminary results highlight that the H.O.M.E. VR‐based intervention protocol could benefit from some revision in order to better target psychological inflexibility and experiential avoidance more consistently. The lack of VR‐based interventions capable of targeting these two transdiagnostic factors in the prevention of EDs or in other clinical and nonclinical populations (Gardini et al. 2022) does not allow authors to draw conclusions on specific aspects that could be improved in H.O.M.E. VR‐based intervention protocol and highlights the necessity of incorporating qualitative evaluations from both participants and clinicians using the H.O.M.E. software and intervention. These qualitative evaluations will help to identify areas for improvement, refine the intervention's design and further explore its effects on transdiagnostic as well as ED‐related factors. A higher number of sessions could also help produce greater changes in this aspect, as traditional psychotherapeutic interventions in similar populations usually entail a longer duration (Manlick, Cochran, and Koon 2013).

In line with recommendations for developing and testing VR intervention protocols and clinical trials (Birckhead et al. 2019), this preliminary study is also aimed at comparing the effects achieved by the H.O.M.E. VR‐based intervention to those achieved by a waiting list condition, to establish whether the obtained changes could be attributed to the intervention and thus determine whether it could be tested when used against to or in combination with active traditional psychological interventions (e.g., CBT‐E) as well. Results managed to establish the H.O.M.E. VR‐based intervention promising effects compared to the waiting list. Indeed, all the aforementioned changes on targeted outcomes achieved by the VR group were greater than those achieved by the waiting list group, who also did not report any significant difference between pre‐ and postintervention levels of dysfunctional eating behaviours or transdiagnostic factors. In the literature, VR‐based interventions have often proven their efficacy against nonactive control groups for the treatment of several psychopathologies (including EDs) (Ferrer‐García and Gutiérrez‐Maldonado 2012; Freeman et al. 2017; Geraets, Wallinius, and Sygel 2022; Riva, Malighetti, and Serino 2021). Although to date no preventive VR‐based interventions are available for EDs specifically, the results achieved by the H.O.M.E. intervention can pave the way towards future studies to further confirm the potential benefits of this protocol and VR as a technology for the prevention of these psychopathologies, especially when also targeting transdiagnostic factors and when compared to or used in addition to traditional psychological interventions.

Moreover, the potential preventive utility of H.O.M.E. was also proven by the maintenance of the achieved improvements at follow‐ups of 3 and 6 months. Indeed, while participants in the waiting list condition seemed to experience the same levels of symptoms after 3 months and even a worsening of dysfunctional eating behaviours and ER difficulties after 6 months, improvements achieved by the VR group remained unchanged in the months following the intervention. Although longer follow‐ups are needed to further prove the maintenance of these results, they confirm the potential of H.O.M.E. and VR in general to make long‐lasting positive changes in the GP at risk for EDs.

Another important and promising element to underline is that no participant dropped out during the H.O.M.E. intervention and the entirety of the VR group underwent all the six sessions. This is of clinical relevance as drop‐out rates in treatments targeting people with EDs are high (Dalle Grave 2014) and motivation of participants undergoing ED prevention protocols can be low (Harrer et al. 2020; Shaw, Stice, and Becker 2009): the interactive component of VR may have indeed contributed to engaging participants in completing the intervention, as it has oftentimes been observed in the literature (Riva et al. 2016).

### 4.1 Limitations

The promising results achieved by the presented study should be observed in light of the methodological limitations, such as the small sample size, which might be due to participants' personal availability to take part in the study, and also to the egosyntonic nature of ED symptoms which may reduce people's motivation to change (Roncero et al. 2013). The small sample size, as well as its homogeneous characteristics (that is, GP at risk for EDs and predominantly young female university students), also limits the generalisability of these potential results to larger populations, other age and gender groups, and clinical samples with a full ED or other DSM‐5‐TR diagnoses. Future studies already plan to test the H.O.M.E. intervention protocol in more heterogeneous and clinical ED population to further test its potential in the prevention and treatment of EDs. The use of a waiting list condition as a nonactive control group for comparison also represents a limitation in assessing the true efficacy and effectiveness of the H.O.M.E. VR‐based intervention protocol. However, these promising results also pave the way to future research testing H.O.M.E. when used against or in addition to evidence‐based treatments (e.g., CBT‐E).

Moreover, the self‐report measures used for the evaluation of the variables include several limitations, such as the tendency participants may have to answer questions based on social desirability and the difficulties people may encounter in answering questions with a reliable level of insight, especially when they concern complex psychological factors such as psychological inflexibility and experiential avoidance (Chan 2008). Moreover, the MPFI‐EA (Landi et al. 2021; Rolffs, Rogge, and Wilson 2018), used to evaluate experiential avoidance in the study, consisted of only five items, which may have led to methodological biases. Studies evaluating participants' opinions about the H.O.M.E. software and intervention using semistructured interviews are being conducted in order to better assess the overall quality of H.O.M.E. as well as its impact on a more subjective level.

Finally, the fact that the H.O.M.E. software needs to be used at the presence of a clinical psychologist guiding the patient through the environment allowed the intervention to be tailored to the participants' needs, but it also limits the applicability of the intervention outside of the psychologist's office. Therefore, authors also plan to develop a standalone version of the H.O.M.E. protocol using different technologies, such as augmented reality or mobile apps, which will allow the H.O.M.E. VR‐based intervention to be offered to an even wider range of people (Cipresso et al. 2018; Riva et al. 2016; Vinci et al. 2020), thus making the protocol more accessible and useful in preventing the onset of EDs and other mental health conditions in more individuals at risk.

### 4.2 Conclusion

While there are numerous studies in the literature that attest to the usefulness of VR systems in the treatment of EDs (Ferrer‐García and Gutiérrez‐Maldonado 2012; Riva, Malighetti, and Serino 2021), those investigating the use of VR‐based interventions in the prevention of EDs in the GP at risk, especially through the improvement of transdiagnostic factors, are lacking (Ciao, Loth, and Neumark‐Sztainer 2014).

These preliminary results highlighted the potential role that the H.O.M.E. VR‐based transdiagnostic intervention protocol could play in the prevention of EDs, as it reduced dysfunctional eating behaviours, such as preoccupation with eating, shape and weight, and improved some of the transdiagnostic factors that can lead to EDs, in particular difficulties in ER and psychological inflexibility (Levin et al. 2014; Morton et al. 2020; Prefit, Cândea, and Szentagotai‐Tătar 2019).

The present exploratory study also showed good feasibility of the H.O.M.E. protocol and its applicability in bigger and more robust research protocols as well as in the clinical practice. This could prove to be beneficial since, despite the use of VR is gradually spreading in clinical psychology and, in particular, in the assessment, treatment and prevention of EDs (Brown et al. 2020; Rizzo, Thomas Koenig, and Talbot 2019), many issues still hinder the applicability of this technology, such as the high costs (in terms of software, hardware and training) and the predominantly disorder‐specific nature of VR software and intervention protocols available to date (Brown et al. 2020; Emmelkamp and Meyerbröker 2021; Freeman et al. 2017; Gardini et al. 2022). Working on developing and testing transdiagnostic VR software such as H.O.M.E. would improve VR applicability in the clinical practice and in the prevention of psychiatric disorders.

## Conflicts of Interest

The authors declare no conflicts of interest.

## Supporting information


**Table S1** Sociodemographic, clinical characteristics and differences between the group that accepted to participate in the research and the group that refused.
**Table S2** Means and standard deviations of levels of dysfunctional eating behaviours (EDE‐Q) and transdiagnostic factors (DERS‐16, AAQ‐II and MPFI‐EA) at each assessment point for VR and control (waiting list) groups.

## Data Availability

The raw data supporting the conclusions of this article will be made available by the authors, without undue reservation.
