# Supplementary material for: A Novel Transdiagnostic Approach to the Prevention of Eating Disorders Using Virtual Reality: Preliminary Evaluation of the H.O.M.E. Intervention
Source: Clin Psychol Psychother. 2025 Feb 3;32(1):e70040. doi: 10.1002/cpp.70040 (PMC11789710; doi:10.1002/cpp.70040)
Supplement: Supplementary file 1 — Table S1 Sociodemographic, clinical characteristics and differences between the group that accepted to participate in the research and the group that refused. Table S2 Means and standard deviations of levels of dysfunctional eating behaviours (EDE‐Q) and transdiagnostic factors (DERS‐16, AAQ‐II and MPFI‐EA) at each assessment point for VR and control (waiting list) groups. [file CPP-32-e70040-s001.docx]

Supplementary Material

# Supplementary Data

None.

# Supplementary Figures and Tables

**Supplementary Table 1.** Sociodemographic, clinical characteristics, and differences between the group that accepted to participate in the research and the group that refused

|  | Accepted to participate (n=40)  M±SD  *or* % (n=) | Did not participate  (n=64)  M±SD  *or* % (n=) | t(df) | χ^2^_(df)_ | p |
| --- | --- | --- | --- | --- | --- |
| Gender | 100% (n=40) females | 100% (n=64) females | - | - | - |
| Age | 24.55±7.61 years | 23.6±8.08 years | -.59_(102.0)_ | - | .556 |
| Marital status | 90% (n=36) single  5% (n=2) married  5% (n=2) divorced | 92.19% (n=59) single  1.56% (n=1) married  1.56% (n=1) living with partner  4.69% (n=3) divorced | - | 1.65_(3)_ | .648 |
| Educational level | 40% (n=16) high school diploma  57.50% (n=23) university degree  2.50% (n=1) professional school diploma | 53.13% (n=34) high school diploma  45.31% (n=29) university degree  1.56% (n=1) middle school diploma | - | 3.84_(3)_ | .279 |
| Occupation | 72.50% (n=29) university students  15% (n=6) employed  2.50% (n=1) unemployed  10% (n=4) other | 79.69% (n=51) university students  15.63% (n=10) employed  4.69% (n=3) unemployed | - | 6.88_(4)_ | .142 |
| SCOFF | 3.60±1.01 | 3.33±.99 | -1.35_(102.0)_ | - | .180 |
| EDE-Q-Total | 3.33±1.12 | 3.20±1.54 | -.47_(102.0)_ | - | .638 |
| EDE-Q-Restraint | 2.77±1.52 | 2.68±1.79 | -.26_(102.0)_ | - | .795 |
| EDE-Q-Eating concerns | 2.53±1.38 | 2.65±1.67 | .41_(102.0)_ | - | .685 |
| EDE-Q-Shape concerns | 4.39±1.10 | 3.97±1.62 | -1.46_(102.0)_ | - | .147 |
| EDE-Q-Weight concerns | 3.63±1.28 | 3.48±1.75 | -.47_(102.0)_ | - | .640 |

Abbreviations. EDE-Q: Eating Disorders Examination Questionnaire; M: mean; SD: Standard Deviation

**Supplementary Table 2.** Means and standard deviations of levels of dysfunctional eating behaviors (EDE-Q) and transdiagnostic factors (DERS-16, AAQ-II, and MPFI-EA) at each assessment point for VR and control (waiting-list) groups.

|  | *H.O.M.E. VR group (n=20)*  *M±SD* | | | | *Waiting-list group (n=20)*  *M±SD* | | | |
| --- | --- | --- | --- | --- | --- | --- | --- | --- |
|  | **T0** | **T1** | **T2** | **T3** | **T0** | **T1** | **T2** | **T3** |
| *EDE-Q-Total* | 3.48±1.07 | 2.89±1.02 | 2.81±1.41 | 2.86±1.21 | 3.18±1.18 | 3.31±1.12 | 3.21±1.11 | 3.49±1.05 |
| *EDE-Q-Restraint* | 2.79±1.68 | 2.22±1.19 | 2.37±1.41 | 2.30±1.27 | 2.75±1.38 | 2.85±1.27 | 2.77±1.44 | 3.33±1.23 |
| *EDE-Q-Eating concerns* | 2.69±1.44 | 2.20±1.22 | 1.98±1.58 | 2.00±1.35 | 2.36±1.33 | 2.46±1.35 | 2.34±1.15 | 2.55±1.14 |
| *EDE-Q-Shape concerns* | 4.52±.93 | 3.74±1.26 | 3.65±1.81 | 3.71±1.53 | 4.27±1.26 | 4.33±1.31 | 4.25±1.38 | 4.36±1.37 |
| *EDE-Q-Weight concerns* | 3.92±1.11 | 3.38±1.18 | 3.25±1.68 | 3.41±1.37 | 3.30±1.40 | 3.59±1.45 | 3.50±1.47 | 3.70±1.32 |
| *DERS-16-Total* | 47.45±15.77 | 41.20±16.20 | 38.75±17.12 | 37.45±16.45 | 46.95±18.42 | 47.75±15.13 | 47.25±15.34 | 52.30±12.76 |
| *DERS-16-C*larity | 5.75±2.34 | 4.90±2.17 | 5.00±2.70 | 5.20±2.55 | 5.15±2.76 | 5.60±2.23 | 5.40±2.30 | 6.00±2.15 |
| *DERS-16-G*oals | 10.35±3.30 | 9.10±3.68 | 8.30±3.44 | 7.85±3.31 | 9.80±3.82 | 10.30±3.47 | 10.30±3.34 | 11.40±2.70 |
| *DERS-16-I*mpulse | 7.55±3.96 | 6.50±4.19 | 6.20±3.58 | 5.95±3.47 | 7.95±3.76 | 7.75±3.09 | 7.45±3.17 | 8.30±2.90 |
| *DERS-16-S*trategies | 14.70±5.56 | 12.30±5.20 | 12.10±6.21 | 11.45±6.05 | 15.10±6.50 | 15.00±5.91 | 15.10±5.86 | 16.70±4.95 |
| *DERS-16-N*onacceptance | 9.10±3.14 | 8.40±3.49 | 7.15±3.41 | 7.00±3.28 | 8.95±3.94 | 9.10±3.26 | 9.00±3.26 | 9.90±2.61 |
| *AAQ-II* | 28.15±9.56 | 24.35±10.43 | 23.75±11.88 | 24.30±11.40 | 25.85±10.97 | 27.25±10.13 | 27.15±10.01 | 29.10±9.55 |
| *MPFI-EA* | 3.28±.99 | 3.40±1.12 | 3.08±1.28 | 3.00±1.18 | 3.34±1.10 | 3.79±1.08 | 3.54±1.03 | 3.65±1.06 |

Abbreviations. AAQ-II: Acceptance and Action Questionnaire-II; DERS-16: Difficulties in Emotion Regulation Scale, short version; EDE-Q: Eating Disorders Examination Questionnaire; M: mean; MPFI-EA: Multidimensional Psychological Flexibility Inventory-Experiential avoidance scale; SD: Standard Deviation
